# Oral-Health-Related Quality of Life among Non-Syndromic School-Age Children with Orofacial Clefts: Results from a Cross-Sectional Study in Northern Italy

**DOI:** 10.3390/children9071098

**Published:** 2022-07-21

**Authors:** Patrizia Defabianis, Cesare Cogo, Stefania Massa, Federica Romano

**Affiliations:** Department of Surgical Sciences, C.I.R. Dental School, University of Turin, 10126 Turin, Italy; cesare.cogo@edu.unito.it (C.C.); stefania.massa96@gmail.com (S.M.); federica.romano@unito.it (F.R.)

**Keywords:** cleft, children, COHIP, oral-health-related quality of life

## Abstract

The aim of this cross-sectional study was to determine the influence of orofacial clefts on the oral-health-related quality of life (OHRQoL) in a group of Italian children and adolescents and to examine whether gender, age, cleft type, and surgical protocol were associated with patients’ OHRQoL. A total of 71 patients with cleft lip and/or cleft palate (CLP) and 71 age- and gender-matched controls (aged 8 to 18 years) were asked to complete the Child Oral Health Impact Profile (COHIP), a validated and reliable questionnaire to assess self-reported OHRQoL in children and teenagers. Children with orofacial clefts showed statistically significant lower quality of life scores than controls for total OHRQoL and for each of the subscales. Gender, the type of cleft, and the type of surgical protocol had no significant influence on OHRQoL. The negative impact of CLP on the area of self-image was greater in 12–18-year-olds, indicating a higher need for psychosocial counselling. These findings suggest that Italian CLP children and adolescents experience a poorer OHRQoL in comparison to their non-cleft peers.

## 1. Introduction

Orofacial clefs are the most common craniofacial malformations caused by anomalies in development and the fusion of facial processes during embryogenesis due to environmental and genetic factors [1]. They are classified as “syndromic” if the malformation is part of a wider condition in a recognizable pathologic pattern or as “non-syndromic” if it appears as an isolated defect or if syndromes cannot be identified [2]. The frequency of cleft lip and cleft palate (CLP) is higher in males and is increased in Asia and Indo-America (1/500) compared to Europe (1/1000) and Africa (1/2500), while the prevalence of cleft palate (CP) is twice as high in females compared to male subjects and uniform among ethnic groups, being approximately 0.5 per 1000 live births [3,4,5]. Unilateral cases of cleft lip (CL) occur more often on the left side [5]. In Italy about 1.03/1000 children are born with a facial cleft [6]. 

Orofacial clefts are associated with numerous complications that alter the patient’s quality of life (QoL) in the physical, functional, and psychological domains by affecting feeding, speech, hearing, and aesthetics [7,8,9,10]. Their management is complex and requires a multidisciplinary corrective approach from the neonatal age until adulthood [11]. Indeed, cleft children undergo continuous surgical treatment, dental care, and speech therapy, which, especially in adolescence, may have psychosocial implications in their daily life, well-being, and social/familiar interaction [12]. Aesthetic concern and speech impairment are often the reason why these patients fail to develop the social skills typical for their age [13]. The most frequent difficulties are related to dental anomalies, constricted maxillary arch form, oronasal fistulas, and velopharyngeal incompetence [14,15,16].

The concept of quality of life related to oral health (oral-health-related quality of life, OHRQoL) is a relatively new notion that encompasses the perception of the actual oral health, focusing on the interaction between oral health, general health, and the related quality of life [17]. According to Yewe-Dyer [18] and Dolan [19] oral health is a disease-free condition in which a comfortable and functional dentition allows an individual to pursue their role within society.

The measurement of OHRQoL in cleft children requires appropriate tools whose results reflect their real needs and the effectiveness of treatments [20]. Recent systematic reviews identified the Child Oral Health Impact Profile (COHIP) as one of the most valid and reliable instruments among those specifically designed for children and teenagers with orofacial anomalies to measure their own OHRQoL [21,22]. Numerous studies have investigated the influence of CLP on OHRQoL using this survey instrument, and they have reported contradictory findings, as some described low levels of QoL, while others did not show any significant impact [23,24,25,26,27]. Differences in age, ethnicity, sample size, treatment protocols, and study designs may partly account for this discrepancy. Scanty data, mostly relying on the use of generic questionnaires, are available from Italy [28].

Therefore, the aim of this cross-sectional study was to determine the OHRQoL in a group of Italian children and adolescents with CLP compared to healthy controls using the COHIP questionnaire. An additional aim was to examine whether gender, age, cleft type, and treatment were associated with patients’ OHRQoL. We hypothesised that children and adolescents with CLP exhibit a similar level of OHRQoL as non-cleft peers. Knowing children’s healthcare needs could contribute to promoting changes in healthcare practices and treatment strategies aimed at improving their well-being.

## 2. Materials and Methods

The present mono-centre observational study was conducted at the Section of Paediatric Dentistry, University of Turin (Italy) from November 2019 to January 2022 after the approval of the Institutional Ethical Committee of the “AOU Città della Salute e della Scienza” of Turin (n. 0038526) and in accordance with the Helsinki Declaration. Written informed consent was received from the parents or legal guardians of all participants. After enrolment, subjects were assigned a number in order to guarantee their anonymity throughout the study.

### 2.1. Participants

All children and adolescents aged between 8 and 18 years, both genders, and suffering from non-syndromic unilateral or bilateral CL, unilateral or bilateral CLP, or CP were invited to participate during the routine follow-up visits. They regularly attended the Plastic Surgery Division of Regina Margherita Hospital (Turin, Italy) and had undergone corrective surgery consisting of either early periosteal palate plastic treatment (EPP) or delayed palate plastic treatment (DPP). The EPP consisted of one-stage palatoplasty combined with lip and soft palate repair at 2–5 months of age. The DPP included infant orthopaedics using a Hotz neonatal plate, cheiloplasty at the age of 3–6 months, and soft palate repair at 8–10 months. Closure of the cleft in the hard palate was carried out at 4 years with a mucoperiosteal flap [16]. The exclusion criteria were subjects with intellectual disabilities and any syndrome or associated craniofacial malformation.

Healthy controls matched for age and gender to CLP subjects were randomly identified from the dental hospital database and were recruited when attending their scheduled follow-up appointments.

### 2.2. Data Collection and Questionnaire

The following data were collected from medical records: age, gender, ethnicity, the presence of any concomitant systemic pathology, the type and side of the cleft, and the type and time of the surgical corrective protocol. All participants were asked to complete the COHIP questionnaire on the same day as their routine appointment [29]. A preliminary trial was carried out to verify the cultural adaptation of the Italian version prior to the start of the present study, involving 30 CLP patients and healthy controls aged 8 to 18 years.

The COHIP contains 34 items divided into five subscales: oral health (the perception of oral symptoms such as pain and tooth sensitivity); functional well-being (trouble with daily life activities such as speaking clearly or chewing); social/emotional well-being (impact on emotional status and socialization); school environment (tasks associated with the school environment), and self-image (sense of self).

The questions were presented directly to participants supervised by a medical observer. They were asked to answer each question regarding his/her experience within the previous 3 months using a five-point rating scale (from 0 = never to 4 = almost all of the time), with another additional response option of ‘I don’t know’ (DK) that was set to missing. If the participants did not answer at least 75% of the items, their questionnaires were excluded from the analysis [29]. If a subscale had missing responses in more than two thirds of the items, the subscale and the overall score were set to missing [29]. The negatively formulated items were reverse-coded before analysis so that higher values of the total COHIP and the subscales reflected a more positive OHRQoL. The OHRQoL score was calculated by adding up the subscale scores, with a total score ranging from 0 to 136.

### 2.3. Statistical Analysis

Accepting an alpha error of 0.05 and a power of 80%, 71 subjects per group were required to detect a difference equal to or greater than 14.0 points on the total COHIP score, based on the results of a previous study [29].

The statistical analysis was performed using the Statistical Package for the Social Sciences (SPSS), version 24.0 software (Chicago, IL, USA). Quantitative variables were summarised using means, medians, interquartile ranges (IQRs), and standard deviations, while categorical variables were summarised using frequencies and percentages. The frequency of responses was analysed for each item and domain of the COHIP questionnaire. The internal consistency reliability was confirmed by Cronbach’s alpha coefficient.

The COHIP total score and five subscale scores of cleft children were compared with those reported by non-cleft controls. The impacts of gender (boy or girl), age (8–11 years or 12–18 years), the type of cleft (CLP, CL, or CP), and the type of corrective surgical intervention (EPP or DPP) were also analysed. A Mann–Whitney *U* test was applied to compare two independent samples, while a Kruskal–Wallis test was used to compare three or more independent samples, followed by post hoc analyses. The association between qualitative variables was determined with the chi-square test. The level of significance was set at 5%.

## 3. Results

A total of 142 subjects, 71 patients with CL, CLP, or CP (mean age 11.91 ± 2.74 years) and 71 control patients (mean age 11.87 ± 2.65 years), were enrolled in the study. The median age was 12. Both groups included 31 females (43.7%) and 40 males (56.3%); most of them were Caucasian (76.1 versus 70.4%). The majority of the cleft subjects had CLP (12.7% bilateral and 61.9% unilateral). The remainder had either CL (12.7%) or CP (12.7%). Forty-six children had been treated with DPP at Regina Margherita Hospital, sixteen had been treated with EPP in other cleft centres in Italy. No information about the type of cleft surgery was available for nine subjects (Table 1).

Within the CLP sample, the frequency of DK response was 0.5%, whereas all control subjects answered each question of the COHIP. All items had less than 5% missing scores. The internal consistencies of the COHIP for cleft patients and controls were good, with Cronbach’s alphas of 0.861 and 0.880, respectively.

Table 2 shows a comparison of the overall and subscale scores for the cleft and control groups. The cleft group presented significantly worse values compared to the control group for the overall COHIP (96.4 ± 18.1 versus 117.6 ± 10.2, *p* < 0.001) as well as in all the dimensions of the questionnaire. 

In order to assess possible age-related differences, participants were split into two groups (8 to 11 years and 12 to 18 years). As reported in Table 3, there were still statistically significant differences between the cleft and control subjects in both the 8-11 and 12–18 age groups in the overall and subscale scores (all *p* < 0.001). 

When data were stratified by gender (Table 4), both females and males with CLP scored significantly lower than female and male non-cleft peers in the subscales “oral health”, “functional well-being”, “social emotional”, and “school environment”. In the dimension of “self-image”, females with CLP scored worse than non-cleft females (*p*: 0.026), while males reported similar scores as their peers. 

Considering only the cleft subjects, Table 5 summarises the intragroup comparison of the overall and subscale scores according to gender, age, cleft classification, and the type of reconstructive surgical protocol. Gender, the type of cleft, and the type of surgical protocol had no effect on OHRQoL. Age differences were found only on the subscales “social emotional” and “self-image”, with subjects aged between 12 and 18 years scoring lower compared to cleft children aged between 8 and 11 years (*p*: 0.053 and *p* < 0.001). 

## 4. Discussion

The present study compared the OHRQoL of Italian children and adolescents with orofacial cleft with that of age- and gender-matched controls using a validated and reliable instrument that was specifically designed for them [29]. The COHIP provides a more comprehensive insight into the OHRQoL than generic questionnaires since it assesses relevant issues for the cleft patient population [30]. The internal consistencies of the overall scale for both cleft and control children were higher than 0.80, and thus they were rated as good according to Broder and Wilson-Genderson [29]. 

The present results indicate a negative impact of CLP on OHRQoL. Therefore, the null hypothesis was rejected. Cleft children had significantly worse scores than controls for the overall COHIP and its dimensions, with oral health and functional and social-emotional well-being as the most impacted domains. These findings are consistent with those from other studies that applied the COHIP tool [23,27,29]. Broder and Wilson-Genderson found in the US that craniofacial patients had significantly lower values for the total COHIP and the functional well-being and school environment domains compared to orthodontic and paediatric patients, while the differences in the social-emotional well-being area were close to statistical significance [29]. Ward et al. [23] observed significantly lower scores for the functional and social-emotional well-being scales in a sample of American CLP children, and Ali et al. found that CLP had some adverse impacts on both the overall and subscales of COHIP among a group of Sudanese children [27]. In contrast, Aravena et al. [24] observed similar OHRQoL among Chilean children with and without orofacial cleft, and Nolte et al. [25] reported relatively high scores among Dutch cleft children comparable to those of parents/caregivers, indicating a good OHRQoL. Similar results were obtained in Thailand [31]. This discrepancy may be attributed to differences in the sample sizes, study populations (socioeconomic and cultural status and ethnic/racial oral health disparities), and ages of participants, as mentioned in a previous study [29]. Moreover, slightly different forms of the COHIP were used. 

When analysing which individual COHIP item showed the highest differences between the CLP and control subjects, we observed that for the oral health and functional well-being subscales, CLP subjects were more likely to have crooked and spaced teeth, to experience food impaction, and to have difficulty keeping their teeth clean and being understood when speaking. Difficulties with oral hygiene are consistent with the dental anomalies associated with orofacial clefts. Tooth malposition, crowding, rotation along with collapsed maxillary arch, and skeletal discrepancies are highly prevalent among children with CLP [14,15,16]. In a recent study from Italy, CLP subjects scored higher on the Oral Health Impact Profile (OHIP)-14 questionnaire than non-cleft peers after a tailored oral hygiene program [28]. Factors contributing to poorer speech can be velopharyngeal dysfunction and oronasal fistulas, which result in a nasal tone and reduced intraoral pressure when pronouncing consonants [32]. 

With regard to the social-emotional well-being subscale, subjects with orofacial cleft were more likely to be anxious or worried about what people think, to feel like they looked different, and to be uncomfortable due to their dissatisfaction with their facial appearance. These findings are in line with those from previous studies [27,33], and they are further supported by the conclusions of a recent systematic review in which speaking, eating, and emotional well-being were found to be the most affected areas of OHRQoL among children and adolescents with CLP [9]. 

When data were stratified by gender, female and male CLP subjects scored worse than their healthy counterparts in all scale dimensions except for self-image. In this domain, females tended to rate lower than their non-cleft peers’, while males did not. This finding is supported by the fact that females generally have more concerns about their body image and they are more vulnerable to cleft repair stigma than males [34]. Consistently, Broder and Wilson-Genderson found lower scores for the emotional well-being domain among females than males, probably because females experienced greater levels of dissatisfaction and anxiety regarding their cleft appearance [29]. Nonetheless, no clear conclusions can be drawn from the literature. Some studies found lower self-esteem among the CLP population [27,28], while others argued that the patients’ acceptance of their appearance lead to a more favourable self-esteem than unaffected children [24,35]. 

The analysis of the impact on OHQoL of clinical factors such as age, gender, the type of cleft, and the corrective surgical protocols provided valuable information. The comparison by cleft type and surgical protocol did not show any significant variation in the overall OHRQoL score nor in the subscales. This finding is supported by earlier studies [26,36] but contrasts with others [10,31,37,38] reporting that children with palatal involvement had lower OHRQoL and, in particular, poorer scores in functional domains, while those with lip involvement were more concerned with psychological aspects. It should be considered that treatment protocols differ according to the types of clefts. In the present study, children underwent either DPP or EPP, and all had completed reconstructive surgeries by the age of four. 

Furthermore, gender did not reveal any significant association with OHRQoL, whereas age did but only with the social emotional and self-image subscales. Indeed, orofacial clefts demonstrated a greater influence on these COHIP dimensions in 12-18-year-olds than they did in 8-11-year-olds, possibly because aesthetic complaints and concerns about the opinions of others tend to increase with age [39]. There are inconsistent results on the effect of age on the OHRQoL of individuals with oral clefts. Some studies found that children and young adolescent groups had similar OHRQoL scores [23,31]. Conversely, others reported that patients aged 12 years or older scored lower in the emotional well-being area than younger children, suggesting that the difference in facial appearance related to lip involvement has a greater importance as the child gets closer to adolescence [10,25,38].

The current study has some limitations. With respect to the application of the COHIP questionnaire, the high number of questions could have influenced the level of comprehension and the concentration of younger children. Regardless, the reliability of the instrument was high and comparable with the coefficient levels reported in the literature [28,30]. The comparison among different types of clefts has to be drawn with some caution due to the small sample size of some cleft groups. Considering that there is no consensus about normal values for CLP patients, the impact of orofacial clefts on OHRQoL was interpreted with respect to the scores of a matched non-cleft sample. Finally, the data refer only to a cleft centre in Northern Italy. This may limit the generalisability of the results.

Further larger studies employing specific and validated tools to assess OHRQoL are required to provide more insight into the perception of individuals with CLP and of their families. Children are stigmatised because of speech disturbance (CP), facial appearance (CL), or both (CLP); thus, they are at higher risk of developing problems in the functional, social, and emotional dimensions [40]. Furthermore, future clinical research should collect information regarding OHRQoL because it can contribute to optimising the rehabilitation process. Examining OHRQoL during different developmental time points is crucial for detecting changes over time and for carrying out appropriate actions according to the children’s ages. The implementation and maintenance of tailored multidisciplinary interventions are fundamental to re-establish aesthetics and function and to enhance the children’s emotion regulation strategies. This may decrease the risk for later psychological problems in such a vulnerable population.

## 5. Conclusions

In the present study, despite the advances in the corrective surgical techniques, oral clefts considerably affected the quality of life of Italian children and adolescents when compared to their non-cleft peers. Interestingly, gender, type of cleft, and surgical protocol had no significant effect on OHRQoL. The negative impact of CLP on the area of self-image was greater in 12–18-year-olds, indicating a higher need for psychosocial counselling in this age range. Therefore, it is crucial to develop psychology-based interventions integrating OHRQoL findings.

## Figures and Tables

**Table 1:** General characteristics of the sample.

Parameter	Cleft Patients (*N* = 71)	Controls (*N* = 71)	*p* Value
Gender (*n*, %)			1.00
Male	40 (56.7)	40 (56.7)	
Female	31 (43.7)	31 (43.7)	
Age group (*n*, %)			1.00
Child (aged 8–11 years)	32 (45.1)	32 (45.1)	
Adolescent (aged 12–18 years)	39 (54.9)	39 (54.9)	
Ethnicity (*n*, %)			0.570
Caucasian	54 (76.1)	50 (70.4)	
Not Caucasian	17 (23.9)	21 (29.6)	
Cleft type (*n*, %)			
CL	9 (12.7)	-	
UCLP	44 (61.9)	-	
BCLP	9 (12.7)	-	
CP	9 (12.7)	-	
Surgical protocol (*n*, %)			
EPP	16 (22.5)	-	
DPP	46 (64.8)	-	
Not available	9 (12.7)		

BCLP; Bilateral cleft lip and palate; CL, cleft lip; CP, isolated cleft palate; EPP; Early palate plastic surgery; DPP; Delayed palate plastic surgery; UCLP, Unilateral cleft lip and palate.

**Table 2:** Overall and subscale COHIP scores by group.

COHIP (Maximum Possible Score)	Group				*p* Value
	Cleft patients (*N* = 71)		Controls (*N* = 71)		
	Mean ± SD	Median (IQR)	Mean ± SD	Median (IQR)	
Overall COHIP (136)	96.4 ± 18.1	101.0 (28.0)	117.6 ± 10.2	121.0 (13.0)	<0.001
Oral health (40)	26.9 ± 5.2	27.0 (6.0)	31.9 ± 4.2	33.0 (6.0)	<0.001
Functional well-being (24)	17.7 ± 3.5	18.0 (4.0)	21.7 ± 2.2	22.0 (4.0)	<0.001
Social emotional (32)	23.9 ± 8.0	26.0 (13.0)	30.1 ± 3.5	31.0 (2.0)	<0.001
School environment (16)	12.4 ± 2.7	13.0 (3.0)	15.1 ± 1.5	16.0 (2.0)	<0.001
Self-image (24)	15.5 ± 5.5	15.0 (10.0)	18.9 ± 3.0	19.0 (6.0)	<0.001

COHIP, Child Oral Health Impact Profile; IQR, Interquartile range.

**Table 3:** Overall and subscale COHIP scores by age and group.

COHIP	Group								*p* Value
	Cleft Patients				Controls				
	8–11 years (*N* = 32)		12–18 years (*N* = 39)		8–11 years (*N* = 32)		12–18 years (*N* = 39)		
	Mean ± SD	Median (IQR)	Mean ± SD	Median (IQR)	Mean ± SD	Median (IQR)	Mean ± SD	Median (IQR)	
Overall COHIP	101.2 ±15.1 ^A^	104.0 (20.1)	92.5 ± 19.3 ^B^	95.0 (29.0)	119.5 ± 7.5 ^A^	121.5 (8.7)	116.0 ± 11.9 ^B^	119.0 (18.0)	<0.001
Oral health	27.0 ± 4.5 ^A^	27.5 (6.0)	26.8 ± 5.7 ^B^	27.0 (8.0)	32.2 ± 3.2 ^A^	33.0 (4.7)	31.8 ± 4.9 ^B^	33.0 (7.0)	<0.001
Functional well-being	17.3 ± 3.7 ^A^	17.0 (4.7)	18.0 ± 3.3 ^B^	19.0 (4.0)	21.6 ± 1.9 ^A^	22.0 (2.7)	21.7 ± 2.5 ^B^	22.0 (4.0)	<0.001
Social emotional	26.0 ± 7.2 ^A^	29.0 (7.7)	22.1 ± 8.4 ^B^	23.0 (18.0)	31.0 ± 1.8 ^A^	32.0 (8.0)	29.3 ± 4.2 ^B^	31.0 (4.0)	<0.001
School environment	12.7 ± 2.2 ^A^	13.0 (9.0)	12.2 ± 3.0 ^B^	13.0 (5.0)	15.3 ± 1.0 ^A^	16.0 (1.0)	14.9 ± 1.8 ^B^	16.0 (2.0)	<0.001
Self-image	18.1 ± 5.0 ^A^	20.0 (7.7)	13.3 ± 4.8 ^B^	14.0 (7.0)	19.4 ± 2.7 ^A^	20.0 (4.0)	18.4 ± 3.2 ^B^	19.0 (6.0)	<0.001

^A^, statistically significant difference compared to male controls (*p* < 0.001); ^B^, statistically significant difference compared to female controls (*p* < 0.001).

**Table 4:** Overall and subscale COHIP scores by gender and group.

COHIP	Group								*p* Value
	Cleft Patients				Controls				
	Male (*N* = 40)		Female (*N* = 31)		Male (*N* = 40)		Female (*N* = 31)		
	Mean ± SD	Median (IQR)	Mean ± SD	Median (IQR)	Mean ± SD	Median (IQR)	Mean ± SD	Median (IQR)	
Overall COHIP	97.8 ± 18.7 ^A^	102.5 (27.2)	94.6 ± 17.4 ^C^	98.0 (31.0)	116.6 ± 9.1 ^A^	119.0 (13.2)	118.8 ±11.5 ^C^	123.0 (15.0)	<0.001
Oral health	27.1 ± 5.8 ^A^	27.0 (7.0)	26.7 ± 4.3 ^C^	27.0 (7.0)	31.5 ± 3.9 ^A^	32.0 (5.0)	32.5 ± 4.5 ^C^	34.0 (5.0)	<0.001
Functional well-being	17.8 ± 3.8 ^A^	18.0 (4.0)	17.6 ± 3.1 ^C^	18.0 (4.0)	21.4 ± 2.3 ^A^	22.0 (3.0)	21.9 ± 2.1 ^C^	22.0 (3.0)	<0.001
Social emotional	24.8 ± 7.3 ^A^	27.0 (10.8)	22.7 ± 8.9 ^C^	24.0 (18.0)	30.1 ± 3.4 ^A^	31.5 (2.0)	30.0 ± 3.6 ^C^	31.0 (2.0)	<0.001
School environment	12.5 ± 2.7 ^A^	13.0 (4.0)	12.3 ± 2.6 ^C^	13.0 (3.0)	15.1 ± 1.1 ^A^	15.5 (2.0)	15.7 ± 2.0 ^C^	16.0 (1.0)	<0.001
Self-image	15.7 ± 4.9 ^B^	15.0 (8.5)	15.2 ± 6.2 ^D^	15.0 (13.0)	18.5 ± 3.2 ^B^	19.0 (6.0)	19.3 ± 2.7 ^D^	20.0 (4.0)	0.003

^A^, statistically significant difference compared to male controls (*p* < 0.001); ^B^, not significantly different compared to male controls (*p* > 0.05); ^C^, statistically significant difference compared to female controls (*p* < 0.001); ^D^, statistically significant difference compared to female controls (*p* < 0.05).

**Table 5:** Overall and subscale COHIP scores in cleft patients by gender, age, type of cleft, and type of surgical protocol.

	COHIP Scores					
	Overall COHIP	Oral Health	Functional Well-Being	Social Emotional	School Environment	Self-Image
Gender						
Male (*N* = 40)	97.8 ± 18.7	27.1 ± 5.8	17.8 ± 3.8	24.8 ± 7.3	12.5 ± 2.7	15.7 ± 4.9
Female (*N* = 31)	94.6 ± 17.4	26.7 ± 4.3	17.6 ± 3.1	22.7 ± 8.9	12.3 ± 2.6	15.2 ± 6.2
*p* Value	0.400	0.296	0.688	0.568	0.930	0.667
Age group						
8-11 years (*N* = 32)	101.2 ±15.1	27.0 ± 4.5	17.3 ± 3.7	26.0 ± 7.2	12.7 ± 2.2	18.1 ± 5.0
12-18 years (*N* = 39)	92.5 ± 19.3	26.8 ± 5.7	18.0 ± 3.3	22.1 ± 8.4	12.2 ± 3.0	13.3 ± 4.8
*p* Value	0.056	0.857	0.214	0.053	0.608	<0.001
Cleft type						
CL (*N* = 9)	89.0 ± 26.5	26.0 ± 7.4	18.1 ± 4.1	19.0 ± 10.2	11.9 ± 3.7	17.8 ± 7.1
R-UCLP (*N* = 13)	95.8 ± 19.2	26.5 ± 6.1	17.1 ± 2.5	23.8 ± 8.0	12.1 ± 3.2	16.3 ± 5.4
L-UCLP (*N* = 31)	96.5 ± 16.4	27.3 ± 4.6	18.2 ± 3.8	23.9 ± 7.2	12.1 ± 2.7	15.1 ± 5.3
BCLP (*N* = 9)	98.6 ± 16.8	26.3 ± 4.2	16.9 ± 2.8	25.0 ± 6.9	13.2 ± 1.4	17.1 ± 5.0
CP (*N* = 9)	102.2 ± 14.5	27.8 ± 5.0	17.3 ± 3.7	27.8 ± 8.6	13.7 ± 1.6	15.7 ± 5.0
*p* Value	0.746	0.927	0.480	0.272	0.607	0.752
Surgical protocol						
EPP (*N* = 16)	100.5 ± 20.0	28.1 ± 7.1	16.7 ± 4.8	26.1 ± 6.8	12.9 ± 2.4	16.6 ± 5.1
DPP (*N* = 46)	95.0 ± 18.2	26.5 ± 4.7	18.1 ± 3.1	23.3 ± 8.7	12.1 ± 2.9	15.1± 5.3
*p* Value	0.544	0.237	0.849	0.698	0.240	0.424

BCLP, Bilateral cleft lip and palate; CL, cleft lip; CP, isolated cleft palate; EPP, Early palate plastic surgery; DPP, Delayed palate plastic surgery; L-UCLP, Unilateral cleft lip and palate on the left side; R-UCLP, Unilateral cleft lip and palate on the right side.

## Data Availability

The dataset used and analysed during the current study is available from the corresponding author on reasonable request.
